# CareerCorpus: A comprehensive dataset of annotated resumes

**DOI:** 10.1016/j.dib.2026.112567

**Published:** 2026-02-11

**Authors:** Md Sagor Chowdhury, Adiba Fairooz Chowdhury, Ayesha Banu, Riad Hossain

**Affiliations:** a Department of Computer Science and Engineering, Chittagong University of Engineering and Technology (CUET), Chattogram, Bangladesh; b Department of Computer Science and Engineering, East Delta University, Chattogram, Bangladesh

**Keywords:** Resume dataset, Resume classification, Natural language processing, Automated recruitment, Text analysis, Open access dataset

## Abstract

The CareerCorpus dataset contains 302 annotated resumes collected from Kaggle (LiveCareer.com) and LinkedIn, covering six occupational categories: Teacher, Finance, Apparel, Accountant, Banking, and Research Assistant. The dataset supports multi-class classification for resume categorization. Each resume received dual annotations from domain experts with verified professional or academic backgrounds in their respective fields. Financial categories (Finance, Accountant, Banking) were annotated by professionals with 5+ years of accounting experience and ICMAB certifications, while specialized categories were annotated by industry practitioners and university lecturers. Data preprocessing involved HTML-to-text conversion using GPT-5, standardized formatting, removal of personally identifiable information (PII), duplicate elimination, and text normalization. Both annotations are preserved in the dataset to enable flexible consensus methods and annotation uncertainty analysis. Inter-annotator agreement varies by category: Apparel (*r* = 0.89), Finance (*r* = 0.68), Research Assistant (*r* = 0.67), Teacher (*r* = 0.56), Banking (*r* = 0.38), and Accountant (*r* = 0.35). Overall mean squared error is 0.023 and mean absolute error is 0.106 across categories. The dataset is released in Excel format (.xlsx) with separate files for each category, available through Mendeley Data under a CC-BY-4.0 license. The dataset can be used for resume classification, skill extraction, recruitment analytics, and related natural language processing research.

Specifications Table

**Table:** 

Subject	Computer Science
**Specific subject area**	Resume classification and automated recruitment analytics using natural language processing; supports six-class fine-grained occupational categorization
**Type of data**	Text Files (xlsx-formatted)
**Data collection**	Resume data were collected from two primary sources: (1) Kaggle dataset containing 2400+ resumes originally scraped from LiveCareer.com, available in both string and HTML formats across 24 job categories, from which we selected 251 resumes from five categories (Teacher, Finance, Apparel, Accountant, Banking); (2) 51 Research Assistant Resumes manually collected from LinkedIn. HTML-formatted resumes were processed using GPT-5 for summarization and text extraction. Each resume was independently annotated by two annotators, with both annotations retained in the dataset to preserve annotation diversity and enable flexibility in downstream applications.
**Data source location**	Institution: Department of Computer Science and Engineering, Chittagong University of Engineering and Technology (CUET), Chattogram, Bangladesh Original data sources: Kaggle (LiveCareer.com resumes), LinkedIn
**Data accessibility**	Repository name: Mendeley Data Data identification number: 10.17632/wzzwn37gmd.1 Direct URL to data: https://data.mendeley.com/datasets/wzzwn37gmd/1 Instructions for accessing these data: The complete CareerCorpus dataset is freely available for download from Mendeley Data under a CC-BY-4.0 license. No registration or authentication is required for access. The repository contains a single Excel file (.xlsx format) with all 302 resumes across six occupational categories (Teacher, Finance, Apparel, Accountant, Banking, Research Assistant), including dual expert annotations and anonymized resume text. The dataset is currently under moderation and will be publicly accessible within 2 business days of submission.
**Related research article**	None

## 1 Value of the Data

• CareerCorpus addresses the limited availability of publicly accessible, expert-annotated resume datasets by providing 302 resumes across six occupational categories with dual human annotations from verified domain experts. Unlike existing datasets that rely on automated labeling or AI-generated annotations, this dataset employs professionals with 5+ years of industry experience (for financial categories) and active academic practitioners (for educational and research categories), offering researchers access to high-quality ground truth labels for training and evaluating resume classification models.
• Researchers can utilize this dataset for multiple natural language processing tasks including multi-class resume categorization, skill extraction algorithms, job-candidate matching systems, and career trajectory analysis. The dual-annotation structure supports research in annotation disagreement modeling, soft-label training, confidence-weighted learning, and human-AI collaboration studies. The dataset's balanced distribution across categories (50–51 instances each) makes it suitable for comparative evaluation of classification algorithms.
• The documented preprocessing methodology provides a replicable template for converting unstructured resume data into research-ready formats. The pipeline demonstrates HTML-to-text conversion using large language models (GPT-5), systematic PII removal procedures, and text standardization techniques that other researchers can adapt for similar dataset development efforts in recruitment analytics or document processing domains.
• The preserved annotation disagreements enable advanced machine learning research that leverages uncertainty quantification and annotation diversity. Researchers can explore consensus-building strategies, develop models that predict annotator confidence, investigate category boundary ambiguities (particularly between overlapping roles like Finance, Accountant, and Banking), and design systems that account for subjective judgment in classification tasks.
• CareerCorpus supports comparative benchmarking and reproducibility in resume analysis research by providing standardized, openly accessible data with transparent annotation procedures. The dataset includes detailed annotator credentials (Table 7) and inter-annotator agreement metrics (Table 8), enabling researchers to assess label reliability and compare their model performance against established baselines using consistent evaluation data.
• The dataset promotes fairness and transparency in automated recruitment research by offering openly accessible data that researchers can independently verify, extend, and audit. The human expert annotation approach and documented category definitions (Section 3) provide a foundation for developing recruitment tools that can be evaluated for bias, tested across different populations, and improved through community collaboration in employment technology research.

## 2 Background

The proliferation of online job platforms and digital recruitment systems has generated an overwhelming volume of resume submissions, creating a pressing need for automated processing solutions. Traditional manual resume screening is time-consuming and prone to human bias, which has driven the adoption of machine learning-based systems. However, the development of such systems is hindered by the limited availability of publicly accessible, well-annotated resume datasets. Existing datasets are often proprietary, limited in scope, or lack standardized annotations [[1], [2], [3]]. To address this gap, CareerCorpus was compiled as a benchmark resource for developing and evaluating resume classification models. By combining professionally crafted resumes from LiveCareer.com with real-world Research Assistant resumes from LinkedIn, CareerCorpus offers diverse resume styles and formats that reflect actual recruitment scenarios. A key challenge in existing resume datasets is the reliance on automated labeling or AI-generated annotations, which can introduce systematic errors, miss domain-specific nuances, and reduce reliability for robust model evaluation. CareerCorpus addresses this limitation by employing dual human expert annotators across all categories. Financial resumes (Finance, Accountant, Banking) were annotated by professionals with 5+ years of accounting experience and formal ICMAB certifications, while specialized categories (Apparel, Teacher, Research Assistant) were annotated by domain experts with direct industry or academic experience. This human-centric approach ensures that subtle distinctions between overlapping categories (e.g., Finance vs. Accountant vs. Banking) are accurately captured, providing a more realistic and challenging benchmark for NLP-driven resume classification research (Table 1).

**Table 1:** Statistical information about the data collection sources.

Source	Affiliation	Resume Count	Format	Quality Criteria
Kaggle/LiveCareer	Teacher, Finance, Apparel, Accountant, Banking	251	HTML, Text	Professional templates, complete sections
LinkedIn	Research Assistant	51	PDF, Text	Public profiles, comprehensive information

## 3 Data Description

Resume screening is a fundamental task in recruitment that significantly influences organizational hiring efficiency and candidate experiences. Currently, online platforms such as LinkedIn, Indeed, and various job portals serve as common channels for job applications, enabling employers and candidates to rapidly communicate. With the increasing number of job seekers and openings, efficient resume processing becomes crucial. Manual screening can lead to delays, biases, and missed opportunities, highlighting the necessity of automated systems to detect qualified candidates and streamline recruitment processes.

This study introduces the CareerCorpus dataset, a corpus focused on resume classification for automated recruitment systems. We compiled a diverse dataset that reflects different professional categories, each showing its own unique language patterns and structural characteristics. To ensure clarity, we carefully defined each category to facilitate accurate understanding and annotation.

### 3.1 Category definitions

To ensure clarity and accurate annotation, we carefully defined each of the six occupational categories:

1. Teacher: Resumes emphasizing educational instruction, curriculum development, classroom management, and pedagogical experience in K-12 or higher education.
2. Finance: Resumes focused on financial analysis, reporting, budgeting, forecasting, and financial management roles in corporate or institutional settings.
3. Apparel: Resumes highlighting fashion design, merchandising, retail management, and product development in the clothing and fashion industry.
4. Accountant: Resumes emphasizing accounting practices, tax preparation, auditing, reconciliations, and financial compliance.
5. Banking: Resumes focused on banking operations, loan management, compliance, risk assessment, and financial services in banking institutions.
6. Research Assistant: Resumes emphasizing academic research experience, publications, laboratory skills, and scholarly activities in research institutions. Each category represents distinct linguistic patterns, skill sets, and career trajectories within their respective domains.

**Fig. 1:**
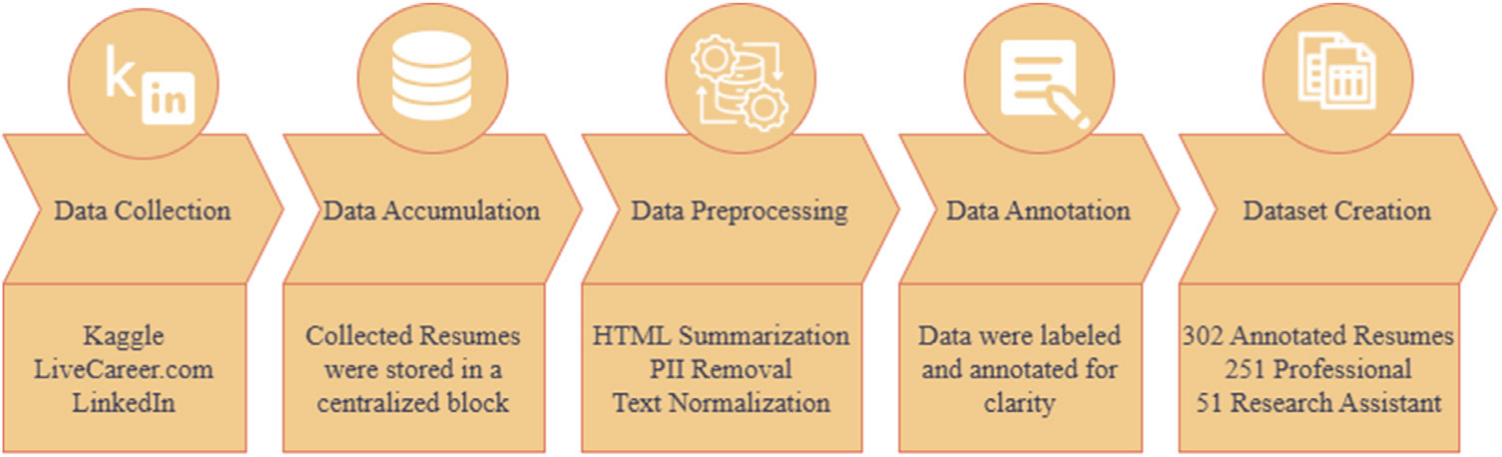
Overall methodology for CareerCorpus dataset development, showing stages from data acquisition to final annotation.

We created the CareerCorpus dataset using established data development methods presented in Fig. 1.

In this section, we discuss data acquisition, the annotation procedure, calculation of annotation agreement, and dataset analysis to provide deeper insights into our developed 'CareerCorpus' dataset.

### 3.2 Dataset acquisition

The dataset employed in this research was gathered from a publicly available Kaggle dataset containing over 2400 resumes originally scraped from LiveCareer.com [4]. This dataset provided professionally crafted resume examples across 24 job categories in both HTML and plain text string formats. We selected five professional categories (Teacher, Finance, Apparel, Accountant, Banking) with approximately 50 resumes each, totaling 251 instances (Teacher: 50, Finance: 50, Apparel: 50, Accountant: 51, Banking: 50). Additionally, we collected 51 Research Assistant resumes from various academic sources, bringing the total dataset to 302 resumes. All HTML-formatted resumes were processed through ChatGPT (GPT-5) to extract structured information and convert them into a standardized text format suitable for annotation and analysis.

### 3.3 Dataset visualization

To visualize the dataset distribution and understand linguistic patterns, we analyzed word frequency and text characteristics across categories. Table 2 presents the most significant keywords from each professional category that help distinguish between different resume types.

**Table 2:** Top keywords for each category.

Category	Top Keywords
Teacher	teacher, university, education, instruction, classroom, management, school, communication, curriculum, senior, grade,support, office, training, math, parent
Banking	Banking, sales, business, management, manager, senior, ops, university, compliance, team, service, leadership, staff, led, risk, training, loan
Finance	finance, reporting, manager, accounting, senior, payroll, financial, ap, bank, audits, cash, management, sales, university, audit, budgeting, business
Accountant	Accountant, accounting, bank, payroll, recs, reporting, cash, tax, reconciliations, senior, management, university, financial, excel, inventory, monthly, analysis
Apparel	apparel, sales, level, manager, senior, training, management, design, customer, merchandising, service, designer, office, team, university, product
Research Assistant	Research, assistant, engineering, level, entry, skilled, university, science, ai, technology, asst, intern, ml

Table 3a, Table 3b present six representative resume profiles from the dataset, split for readability. Each row in Table 3a corresponds to the same ID row in Table 3b, illustrating the complete resume structure including education, skills, experience, and classification labels. The inter-annotator agreement scores in Table 3b range from 0.22–0.99, highlighting varying classification difficulty across job levels and domains.

**Table 3a:** Sample resume profiles—Education and skills component.

ID	Domain	Education	Skills and achievements
26,987,539	Banking	B.L.A., Sociology & Dance — Washington State University. Business & Personal Banker Academy; S.A.F.E. Registered Financial Banker.	Public speaking; MS/Google Suite; KPI-based training; strategic sales facilitation; account management. Trained 600+ employees annually; directed 100+ events; exceeded 794 % of goals ($1.2 M sales in 35 days).
86,549,455	Finance	Ph.D., Organizational Leadership — The Chicago School of Professional Psychology (2013); M.A., Industrial-Organizational Psychology — The Chicago School of Professional Psychology (2008); B.S., Psychology — Belmont University (2006).	Leadership development, employee relations, training & facilitation, project management, predictive/budget planning, data analysis, public speaking, mentoring; developed risk-reduction metrics; board/committee leadership across multiple professional groups.
25,749,150	Accountant	B.A. Accounting, Central Connecticut (2010); Accounting studies, Univ. of Hartford (2016, GPA 3.8);	ICD-10/ICD-9, CPT/HCPCS, EMR, HIPAA, claim entry/payment posting, insurance verification & authorizations, AR/AP, billing, registration, GL/bank recs, variance resolution;
12,467,531	Teacher	M.Ed., Elementary Education & Teaching—UCLA (2016); B.A., Psychology—University of New Mexico (2003); additional study in Studio Arts—Santa Monica College.	Instructional design, standards-aligned planning, course development, progress monitoring, IEP leadership, behavior supports, safe classroom culture, family communication, staff evaluation, project/daily scheduling, process improvement, event planning; tools: Excel, Outlook, Adobe Photoshop, Slack, Evernote; robotics/programming instruction; research, analysis, planning.
24,533,931	Apparel	Completed Business Administration studies at South Louisiana Community College (2018) and University of Louisiana-Lafayette; graduated Salmen High School, 2014	Proficient in sales, cash handling, customer assistance, adaptability, teamwork, communication, and organization; active in 21st Century Summer Camp (2012–2014)
1	Research Assistant	B.*Sc*. in CSE, Chittagong University of Engineering & Technology (GPA 3.83/4, 7th position); HSC, Rajuk Uttara Model College (5.00/5.00); SSC, Uttara High School & College (5.00/5.00)	Skilled in Python, C/*C*++, Java, ML/DL (TensorFlow, PyTorch), NLP; 800+ solved problems; published in EMNLP, NAACL, ACL, JoE; Best Paper (ACL 2023); 1st in CLBLP 2023; 5th–7th at SemEval 2024; 1st in FakeDetect Malayalam; multiple top-10 NLP competition finishes

**Table 3b:** Sample resume profiles—Experience and continuous relevance scores assigned independently by two annotators (Annotator-1 and Annotator-2) *(Continued from*Table 3a*; rows correspond by ID).*

Experience	Job_type	Annotator-1	Annotator-2
Business Banking Specialist (Jun 2014–Present)—sales training, account growth, cross-functional leadership. Personal Banker (Jul 2013–Jun 2014)—relationship management, loan origination. Office & Marketing Manager (Jun 2013–Present)—training programs, event direction, social media. Prior roles in community management, PR, and operations.	Mid-level	0.48	0.8
Finance (Jul 2008–Oct 2015) — roles in integrated scheduling, contracts, business partnership; advised managers, developed metrics, directed training, liaison between leadership and teams; Adjunct Professor (Aug 2013–Present) — teach leadership/human behavior to adult learners and tailor curricula; HR Generalist Intern (Jul 2014–Mar 2015) — bridge management and employees, advise on policy, drive job-description development.	Senior-level	0.49	0.97
Accountant (2012–2014), Medicare Compliance Coder (2010–2012), Hospital Access Rep (2010–2012), Patient Financial Rep (2006–2016);	Mid-level	0.79	0.85
Teacher (08/2014–Present)—designs rigorous lessons, tracks learning, provides timely feedback, leads IEPs, ensures compliance/reporting, partners with families, facilitates group projects, evaluates personnel. Robotics & Design Instructor (08/2012–09/2014)—lectures on kits/programming, leads workshops, adapts curriculum. Executive Advisor (08/2015–Present)—portfolio mgmt (20–25 %/yr over 5 yrs), analytics, relocation, asset restructuring, contingency planning, events (+15 % revenue), office automation, contracts.	Senior-level	0.83	0.95
Over 8 years in retail including Apparel Associate, Cashier/Stocker, and Sales Associate; skilled in merchandising, stock management, and customer service at apparel and housewares departments	Entry-level	0.22	0.42
Lecturer, Dhaka Int’l Univ. (2024–); Software Engg. Intern, Spectrum Engg. Consortium (2023); research mentor & CPC adviser at DIU; developed vehicle scheduling, MERN UI, backend systems	Entry-level	0.9	0.99

**Fig. 2:**
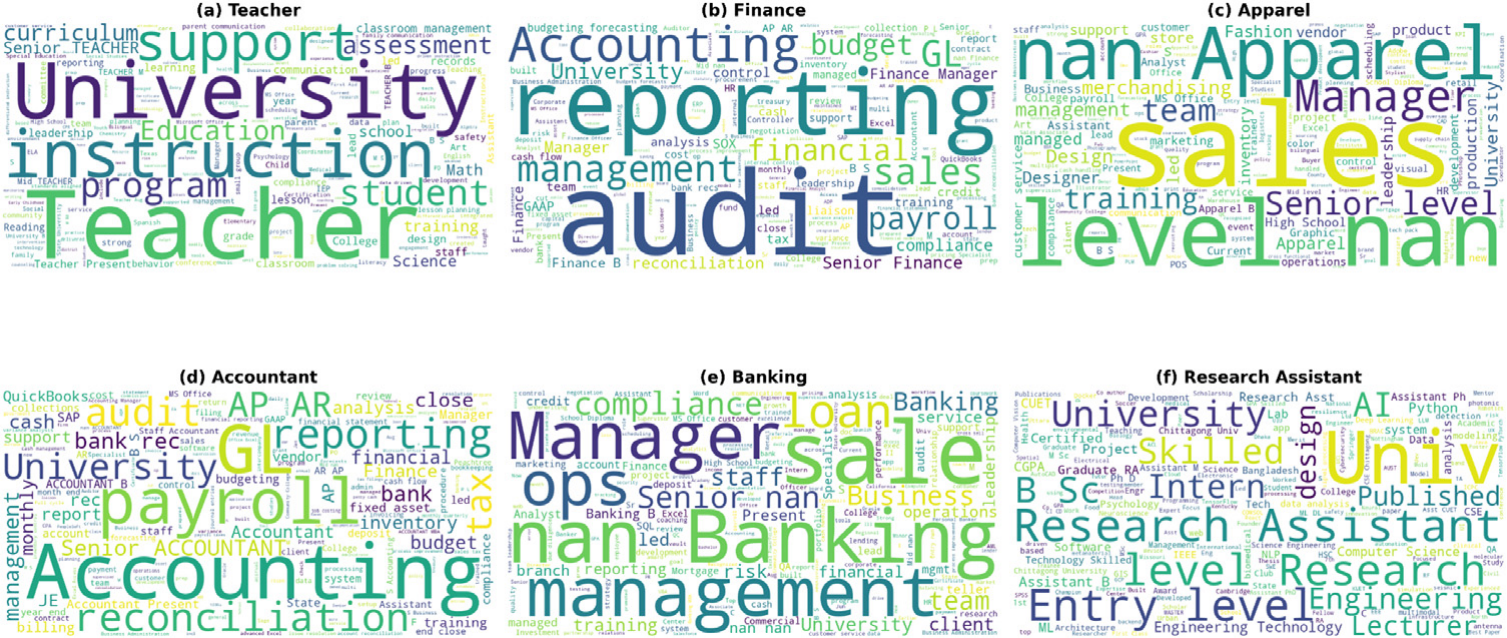
Word cloud visualizations for all six categories in CareerCorpus dataset, showing the most frequently occurring domain-specific terms: (a) Teacher - education and instruction terminology, (b) Finance - accounting and financial reporting terms, (c) Apparel - fashion and merchandising vocabulary, (d) Accountant - technical accounting and compliance terms, (e) Banking - financial services and operations language, (f) Research Assistant - academic research and technical skills.

The values reported under Annotator-1 and Annotator-2 represent continuous relevance scores in the range [0, 1] independently assigned by two domain experts for each resume, indicating the degree of fit between the resume and its assigned occupational category (Fig. 2).

Word clouds were generated using Python's WordCloud library to visualize the most frequently occurring terms in each category. Common stop words and generic terms were removed to highlight distinctive professional vocabulary. The visualizations reveal distinct linguistic signatures for each occupational category: Teacher resumes emphasize pedagogical terminology (“instruction”, “classroom”, “curriculum”), Finance and Accountant categories show technical financial terms (“reporting”, “reconciliations”, “budgeting”), Banking highlights operational and compliance vocabulary (“operations”, “loan”, “risk”), Apparel demonstrates merchandising and design terminology (“merchandising”, “design”, “product”), and Research Assistant resumes feature academic and technical terms (“research”, “AI”, “ML”, “engineering”)

### 3.4 Dataset analysis

Table 4 summarizes the distribution of resumes across the six occupational categories in the CareerCorpus dataset. Most categories contain exactly 50 resumes, while the Accountant and Research Assistant categories contain 51 resumes each, resulting in a near-uniform distribution across classes. This balanced composition is advantageous for multi-class classification tasks, as it minimizes class imbalance and reduces potential bias toward majority categories. The table also provides key statistical characteristics of the dataset that are informative for model development and evaluation.

**Table 4:** Summary of text statistics across different categories.

Category	Total Resumes	Avg. Length (words)	Avg. Sections	Unique Terms
Teacher	50	175	10	1743
Finance	50	183	11	1830
Apparel	50	129	11	1583
Accountant	51	185	12	1665
Banking	50	136	12	1605
Research Assistant	51	126	10	1287
Total	302	156	11	10,713

The lexical analysis shows clear variations in text length and vocabulary across categories. Accountant resumes have the highest average word count (185) and most sections (12), reflecting detailed professional experience and certification requirements. Professional category resumes show moderate consistency in length, with Finance and Banking resumes slightly longer than Teacher and Apparel resumes, suggesting industry-specific documentation requirements (Table 5).

**Table 5:** Sample text entries with their respective categories.

Resume ID	Category	Sample Text Snippet
15,906,625	Accountant	“Government accounting, financial statements, bank recs, AP/AR, fixed assets, payroll, budgeting, closes, tax, GAAP. City of Alexandria Accountant lead AP & fixed assets with statements and bank reconciliations…”
77,156,708	Banking	“Banking Officer with BSA/AML/OFAC compliance, credit analysis and underwriting. Top mortgage originator driving 200 % membership growth. Director of Finance managing budgeting and financial strategy…”
27,789,372	Finance	“Finance Director implemented QuickBooks procedures, prepared payroll and monthly financial statements. Partnered with HR, Compliance, and Treasury. Applied SOX procedures and conducted payroll audits…
8	Research Assistant	“Software Engineer skilled in Python, *C*++, TypeScript, FastAPI, React, Docker, Kubernetes. Top 4 % Codeforces. Developed microservices and migrated Postgres to Spanner. H&M AI Microdegree Scholar…”
27,091,280	Apparel	“Merchandising leadership in women's and children's apparel. Store openings, hiring and training, inventory receiving, floor plans and visuals. Managed high-volume operations exceeding $45 M…”
22,408,666	Teacher	“Classroom management with ELL and Special Education support. After-school program coordinator supervising 7 paraeducators. Coaching volleyball, cheer, and basketball. Bilingual English/Spanish…”

While research on resume classification exists in various forms [[5], [6], [7], [8]], to the best of our knowledge, no existing publicly available dataset specifically addresses comprehensive resume categorization with both professional and academic categories. Table 6 presents a comparative analysis of existing datasets.

**Table 6:** Comparison with existing work.

Reference	Study focus	Approach	Dataset Used	Annotation	Year
[1]	Resume parsing	NLP techniques	Custom	Rule-based	2021
[2]	Resume screening	NLP/ML	Automated	Automated	2023
[3]	Resume analysis survey	NLP	Review paper	Review paper	2024
[5]	ML screening	Machine Learning	Custom	TF-IDF based	2025
[6]	Deep learning screening	LLM-based	1100 resumes	Automated	2025
**Proposed**	**CareerCorpus**	**Multi-class classification**	**302 resumes**	**Human Expert(Dual)**	**2025**

A critical distinction of CareerCorpus compared to existing approaches [[1], [2], [3],^5^,^6^] is the use of comprehensive human expert annotation rather than automated or rule-based labeling. While previous studies primarily relied on keyword matching, TF-IDF scoring, or AI-generated labels, CareerCorpus employs domain-specific expert annotators with verified professional experience. This human-centric approach addresses concerns about bias and fairness in AI recruitment tools [8] by providing high-quality ground truth labels that capture nuanced domain knowledge missed by automated systems [7].

## 4 Experimental Design, Materials and Methods

**Fig. 3:**
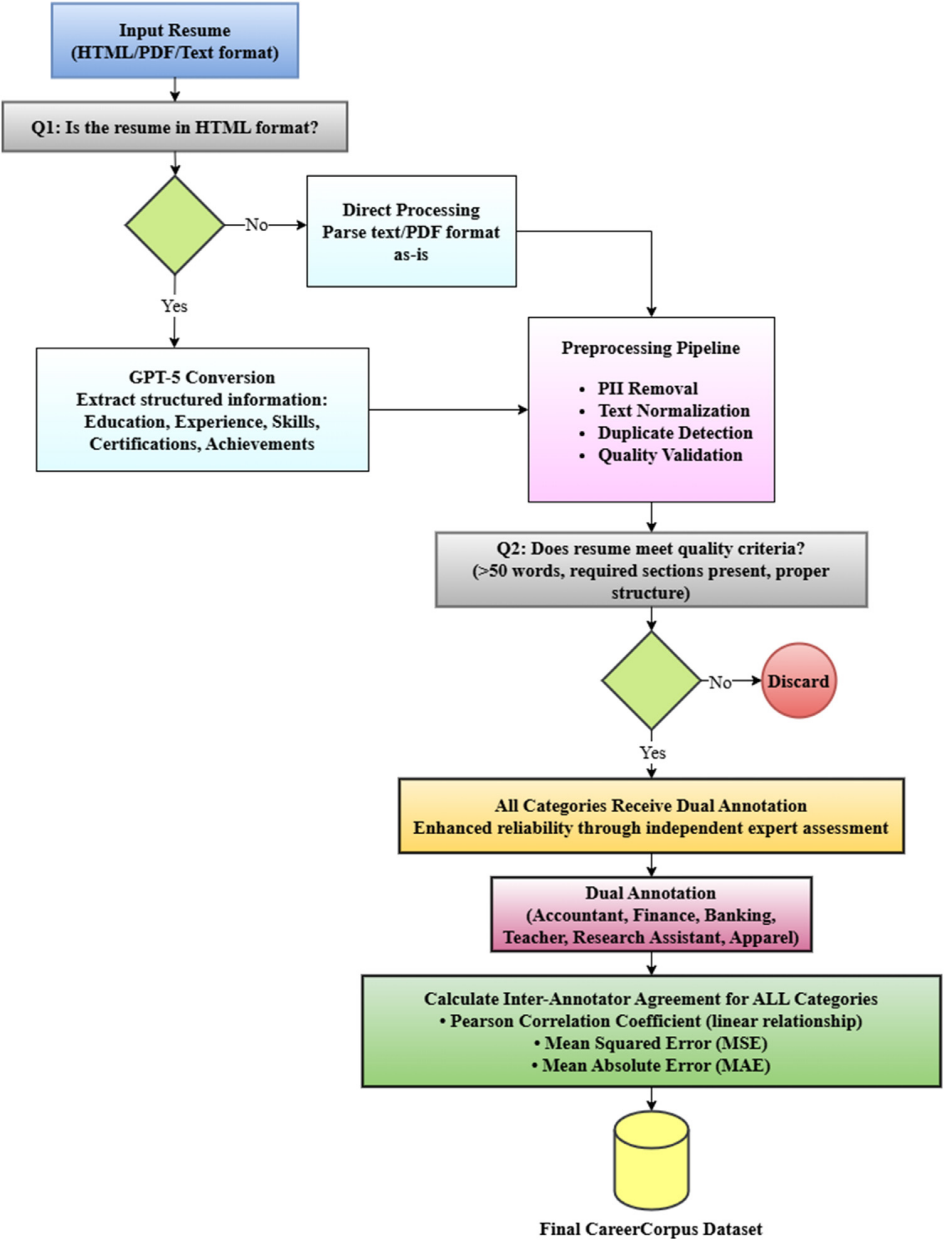
Detailed technical workflow for CareerCorpus dataset development.

This section describes the overall experimental design, including the methods used for data collection, preprocessing, and annotation in the development of the dataset. Fig. 3 presents the structured workflow of the dataset generation process, showing each stage from initial data collection to the final labeling phase.

### 4.1 Preprocessing

Raw resume data often contains structural inconsistencies that can hinder effective model training. To ensure quality and reliability, the CareerCorpus dataset was subjected to a series of preprocessing steps following data collection. First, HTML summarization was performed using ChatGPT (GPT-5), which converted HTML-formatted resumes into structured textual representations. GPT-5 was prompted with carefully designed instructions to extract key information such as education, experience, skills, certifications, and contact details while eliminating HTML tags, formatting noise, and irrelevant metadata. In the second phase, personally identifiable information (PII), including names, addresses, phone numbers, and email addresses, was systematically anonymized or removed to ensure privacy compliance and ethical distribution. Sensitive data was replaced with structured placeholders such as [NAME], [EMAIL], and [PHONE], which preserved logical document flow while protecting identity. Finally, text normalization was applied to standardize formatting across all resumes. This included removing unnecessary characters and whitespace, correcting common spelling variations, normalizing dates and header formats, and ensuring overall textual coherence. These preprocessing procedures collectively produced a refined, uniform, privacy-safe corpus optimized for downstream natural language processing tasks.

### 4.2 Data annotation

Each resume category was annotated by human subject matter experts with relevant domain knowledge and verified professional experience, distinguishing CareerCorpus from existing datasets that rely on automated or AI-generated labels. To ensure quality and consistency, all six categories receive dual annotation by independent experts, allowing for comprehensive inter-annotator agreement calculation and preservation of annotation uncertainty. The annotation scheme was as follows:

• Annotator 1 (An-1): Finance, Accountant, Banking
• Annotator 2 (An-2): Finance, Accountant, Banking
• Annotator 3 (An-3): Apparel
• Annotator 4 (An-4): Teacher, Research Assistant
• Annotator 5 (An-5): Apparel
• Annotator 6 (An-6): Teacher, Research Assistant

This approach provides human expert validation across all categories, ensuring annotation quality that reflects genuine domain expertise rather than automated heuristics or AI-generated labels. The dual-annotation strategy enables robust quality assessment and captures the natural variability in human expert judgment.

### 4.3 Annotation structure

The CareerCorpus dataset provides dual annotations for a total of 302 resumes, with separate scoring columns for Annotator 1 and Annotator 2. This design allows researchers to calculate custom consensus metrics, incorporate annotation disagreement as a feature in model training, evaluate model performance relative to individual annotators, and analyze patterns of variability in human judgment during the annotation process.

### 4.4 Human expert annotation vs. automated labeling

A fundamental design choice in CareerCorpus is the use of human expert annotation rather than automated or AI-generated labels commonly found in existing resume datasets. This decision was motivated by several key considerations:

Domain Expertise: Human annotators bring professional experience and tacit knowledge that automated systems cannot replicate. For example, distinguishing between Finance and Accountant roles requires understanding subtle differences in job responsibilities, reporting structures, and skill requirements that extend beyond keyword matching.

Nuanced Judgment: Resume categorization often involves ambiguous cases where a candidate's background spans multiple domains. Human annotators can assess the primary career trajectory and make contextual judgments based on experience weighting, progression patterns, and stated career objectives—capabilities that exceed current automated classification systems.

Quality Assurance: The dual-annotation framework enables quantitative assessment of label reliability through inter-annotator agreement metrics. This transparency in annotation quality is typically absent in datasets with automated labels, where error rates and systematic biases remain unknown.

Ground Truth Reliability: Machine learning models trained on AI-generated labels may learn to replicate the biases and errors of the labeling system rather than true resume categorization patterns. Human expert annotations provide more reliable ground truth for model training and fair evaluation.

The annotators' professional credentials (documented in Table 7) ensure that labels reflect genuine domain expertise: financial categories are annotated by certified accountants with 5+ years of professional experience, Apparel by industry practitioners, and academic categories by university lecturers with active teaching and research roles. This level of annotator qualification is uncommon in existing resume datasets and represents a significant contribution to dataset quality and research utility.

**Table 7:** Information of annotators.

ID	Academic level	Area of study	Professional Experience	Categories Assigned
An-1	Graduate	Accounting	5+ years as Accountant (SAHIL ENGINEERING & CONSTRUCTION, Merim Co Ltd, BD Design Pvt Ltd); Currently Assistant Manager at Corporate Support Pvt Ltd; Studying ICMAB Professional Level 2	Finance, Accounting, Banking
An-2	Graduate	Accounting & Information Systems (AIS)	Lecturer of Accounting, East Delta University; Former Audit Assistant at ACNABIN Chartered Accountants (Baker Tilly International); Partially qualified Cost and Management Accountant (ICMAB Finalist); Former Accounting Content Creator at 10 Min School	Finance, Accounting, Banking
An-3	Graduate	Textile/Fashion	Lab Incharge at MAS (Apparel company)	Apparel
An-4	Graduate	Education & Research	Lecturer, Department of Computer Science and Engineering, CUET	Teacher, Research Assistant
An-5	Graduate	Textile / Apparel / Industrial Production	Experienced Inspection Manager with a demonstrated history in the apparel and fashion industry; Skilled in textiles, woven fabrics, product development	Apparel
An-6	Graduate	Computer Science & Engineering	Lecturer in Computer Science and Engineering with teaching and research experience (course instruction, research supervision, academic projects)	Teacher, Research Assistant

**Table 8:** Inter-annotator agreement metrics for dual-annotated categories.

Category	Resume Count	Pearson Correlation	MSE	MAE	Interpretation
Finance	50	0.68	0.023	0.103	Moderate positive correlation
Banking	50	0.38	0.062	0.170	Weak positive correlation
Accountant	51	0.35	0.020	0.110	Weak positive correlation
Apparel	50	0.89	0.018	0.121	Strong positive correlation
Teacher	50	0.56	0.012	0.085	Moderate positive correlation
Research Assistant	51	0.67	0.004	0.049	Moderate-to-strong positive correlation
Overall Mean	50	0.59	0.023	0.106	Moderate positive correlation

### 4.5 Annotation procedure

All six categories in CareerCorpus were dual-annotated by independent experts to ensure high-quality labels and to capture variability in human judgment. Both annotations are preserved in the final dataset, enabling researchers to use individual annotator scores for ensemble or probabilistic modeling, derive consensus labels through methods such as averaging or majority voting, study inter-annotator agreement patterns, and train models that account for annotation uncertainty. Explicit annotation criteria were defined based on the category definitions provided in Section 3, and were consistently applied by the annotators throughout the annotation process. The dataset is not intended to be large-scale; rather, it is positioned as a high-quality, expert-annotated benchmark corpus. We explicitly acknowledge dataset size limitations and discuss their implications for deep learning applications in the Limitations section.

### 4.6 Calculation of annotator agreement

Inter-annotator agreement was calculated separately for each occupational category based on the two expert annotations available per resume. Since annotators provided continuous relevance scores rather than categorical labels, we employed multiple quantitative metrics to assess agreement: Pearson correlation coefficient, Mean Squared Error (MSE), and Mean Absolute Error (MAE).

Pearson Correlation Coefficient measures the linear relationship between annotators' scores:

(1) $$r=\frac{\Sigma \left(x_{i}-\underset{‾}{x}\right)\left(y_{i}-\underset{‾}{y}\right)}{\sqrt{\Sigma {\left(x_{i}-\underset{‾}{x}\right)}^{2}\Sigma {\left(y_{i}-\underset{‾}{y}\right)}^{2}}}$$

Mean Squared Error (MSE) quantifies the average squared difference between annotators:

(2) $$MSE\;=\;\frac{\Sigma {\left(x_{i}-y_{i}\right)}^{2}}{n}$$

Mean Absolute Error (MAE) measures the average absolute difference:

(3) $$MAE\;=\;\frac{\Sigma \left|x_{i}-y_{i}\right|}{n}$$

Where $x_{i}$ and $y_{i}$ represent scores from Annotator 1 and Annotator 2 respectively, and n is the number of resumes.

For all categories, both annotations are preserved in the dataset. Researchers can derive consensus labels using averaging (for continuous scores) or majority voting (for categorical labels) based on their specific needs.

Among the financial domains, the Finance category exhibited the strongest inter-annotator agreement (*r* = 0.68), while Banking and Accountant showed weaker correlations (*r* = 0.38 and 0.35, respectively), reflecting greater variability in annotator judgments for these roles. Outside the financial categories, Apparel showed strong agreement (*r* = 0.89), followed by Research Assistant (*r* = 0.67) and Teacher (*r* = 0.56). Error metrics (MSE and MAE) further support these trends: Research Assistant and Teacher categories display relatively lower MAE values (0.049 and 0.085), whereas Banking and Apparel show higher MAE (0.170 and 0.121), indicating greater scoring differences. Overall, the mean correlation across categories was moderate (*r* = 0.59), with an average MAE of 0.106, suggesting consistent but not perfect agreement between annotators for continuous relevance scoring. Cohen’s Kappa was not used in this study, as it is designed for categorical labels and is not appropriate for continuous relevance scoring.

## 5 Dataset Experiment and Benchmark Evaluation

To demonstrate the usability of the CareerCorpus dataset, we conducted lightweight benchmark experiments using standard machine learning models. The purpose was not to propose new algorithms but to verify that the dataset contains meaningful and learnable patterns suitable for automated resume analysis tasks.

### 5.1 Experimental setup

**Fig. 4:**
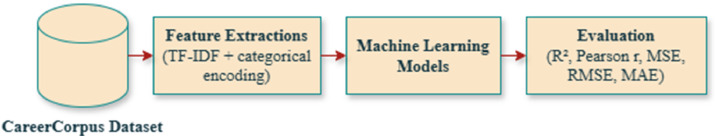
Experimental workflow for evaluating the CareerCorpus dataset.

The average of the two scores was used as the target variable to represent consensus relevance while preserving annotation variability. Textual fields including domain, education, skills and achievements, experience, and job type were combined into a single representation. Features were extracted using TF-IDF vectorization with unigrams and bigrams, supplemented with encoded categorical attributes. The dataset was divided into predefined training and test sets. We evaluated several widely used models, including Ridge Regression, Lasso Regression, ElasticNet, Random Forest, Gradient Boosting, XGBoost, and Support Vector Regression (Fig. 4).

### 5.2 Evaluation metrics

Model performance was assessed using multiple metrics: coefficient of determination (R²), Pearson correlation coefficient (r), Mean Squared Error (MSE), Root Mean Squared Error (RMSE), and Mean Absolute Error (MAE). These metrics jointly measure predictive accuracy and agreement with human annotations.

### 5.3 Results and discussion

Table 9 summarizes the performance of the evaluated models using averaged annotator scores as ground truth.

**Table 9:** Performance of machine learning models on the CareerCorpus dataset.

Model	R2	Pearson r	MSE	RMSE	MAE
Ridge Regression	0.34	0.76	0.018	0.134	0.103
Lasso Regression	0.58	0.80	0.012	0.107	0.087
ElasticNet	0.44	0.76	0.015	0.124	0.101
Random Forest	0.65	0.83	0.0096	0.098	0.073
Gradient Boosting	0.62	0.81	0.010	0.102	0.076
XGBoost	0.63	0.84	0.010	0.100	0.074
Support Vector Regression	0.48	0.86	0.014	0.119	0.105

Ensemble models such as Random Forest and XGBoost achieved the highest performance, indicating that the dataset contains structured and informative patterns suitable for automated resume modeling. Overall, the results confirm that CareerCorpus can support machine learning and natural language processing research while maintaining its primary role as a high-quality annotated dataset.

## Limitations

The CareerCorpus dataset offers valuable insights though it is not without certain limitations. First, the sample s6ze of 302 resumes may be insufficient for training large-scale deep learning models without data augmentation techniques. Second, while the dataset covers six occupational categories, it may not capture the full complexity of real-world resume categorization, particularly for mid-career transitions, hybrid roles, or emerging professions not represented in these categories. Third, resumes from LiveCareer.com represent idealized, professionally crafted examples that may not fully capture the variability, inconsistencies, and formatting issues present in real-world job applications. Fourth, the Research Assistant category comprises only 51 instances, creating class imbalance that may affect model performance and generalization. Fifth, the dataset focuses exclusively on English-language resumes and may not generalize to multilingual or non-English recruitment contexts. Sixth, LinkedIn data was manually collected and may not represent the full diversity of research positions across different disciplines and career stages. Seventh, while human expert annotation provides higher quality labels compared to automated systems, the annotation process is more time-intensive and resource-constrained, resulting in a smaller dataset size (302 resumes) compared to larger automated datasets. However, we argue that annotation quality is more critical than quantity for establishing reliable benchmarks and training robust models, particularly for research applications requiring high-confidence ground truth labels. Finally, while PII has been removed, the original source websites' terms of service regarding data redistribution should be carefully reviewed before commercial applications.While dual annotations provide valuable information about annotation uncertainty, the decision to preserve disagreements rather than resolve them through expert adjudication means that researchers must implement their own consensus strategies. This design choice prioritizes flexibility and transparency over prescriptive label resolution.

## Ethics Statement

The CareerCorpus dataset has been developed following ethical data collection principles. All content was obtained from publicly accessible sources, including the Kaggle repository (originally from LiveCareer.com) and LinkedIn public profiles. The Kaggle dataset [4] was released under an open license permitting research use and redistribution. LinkedIn data was collected exclusively from public profiles where users explicitly made their information publicly viewable. To ensure privacy compliance, all personally identifiable information (PII) including names, contact details, addresses, and any identifying information has been systematically anonymized or removed. The dataset adheres to responsible use principles, emphasizing the protection of individual rights and the prevention of discriminatory practices. Given the sensitive nature of employment data, significant care was taken to ensure fair representation across categories without introducing bias against any demographic group, profession, or educational background. The dataset was independently reviewed by ethics committee members who assessed the content for potential privacy violations or discriminatory patterns. Our primary goal is to support the development of fair and unbiased recruitment automation tools. This work is not intended to replace human decision-making in hiring but rather to augment and improve the efficiency of initial screening processes.

## CRediT authorship contribution statement

**Md Sagor Chowdhury:** Conceptualization, Data curation, Methodology, Formal analysis, Software, Visualization, Project administration. **Adiba Fairooz Chowdhury:** Conceptualization, Data curation, Investigation, Validation, Writing – original draft. **Ayesha Banu:** Data curation, Validation, Resources, Writing – review & editing, Supervision. **Riad Hossain:** Data curation, Validation, Resources, Data curation, Validation, Resources, Writing – review & editing, Supervision.

## Data Availability

Mendeley DataCareerCorpus (Original data).
